# Algorithmic Mechanism Design of Evolutionary Computation

**DOI:** 10.1155/2015/591954

**Published:** 2015-07-16

**Authors:** Yan Pei

**Affiliations:** School of Computer Science and Engineering, The University of Aizu, Aizuwakamatsu 965-8580, Japan

## Abstract

We consider algorithmic design, enhancement, and improvement of evolutionary computation as a mechanism design problem. All individuals or several groups of individuals can be considered as self-interested agents. The individuals in evolutionary computation can manipulate parameter settings and operations by satisfying their own preferences, which are defined by an evolutionary computation algorithm designer, rather than by following a fixed algorithm rule. Evolutionary computation algorithm designers or self-adaptive methods should construct proper rules and mechanisms for all agents (individuals) to conduct their evolution behaviour correctly in order to definitely achieve the desired and preset objective(s). As a case study, we propose a formal framework on parameter setting, strategy selection, and algorithmic design of evolutionary computation by considering the Nash strategy equilibrium of a mechanism design in the search process. The evaluation results present the efficiency of the framework. This primary principle can be implemented in any evolutionary computation algorithm that needs to consider strategy selection issues in its optimization process. The final objective of our work is to solve evolutionary computation design as an algorithmic mechanism design problem and establish its fundamental aspect by taking this perspective. This paper is the first step towards achieving this objective by implementing a strategy equilibrium solution (such as Nash equilibrium) in evolutionary computation algorithm.

## 1. Introduction

Game theory is the methodology used to research strategic interaction among several self-interested agents [1]. Some important concepts, such as* type*,* strategy*, and* utility*, are useful to an understanding of the theoretical framework of game theory. Agent type indicates the preferences of the agent over different outcomes in a game. A strategy is a plan or a rule, which defines the actions that an agent will select in a game. The utility of an agent determines different allocations and payments under its and other agents' types and strategy profiles; for example, an agent rationality in game theory is to implement the expected utility to be maximum. An agent will select a strategy that maximizes its expected utility, given its preferences with regard to outcomes, beliefs about the strategies of other agents, and structure of the game.

Nash equilibrium (NE) is one of the solution concepts that game theory provides to compute outcomes of a game from self-interested agents under certain assumed information that is available to each agent, such as agents types and strategies [2]. It states that every agent in a game should select a maximum utility strategy taking account of other agents' strategies so as to achieve equilibrium. The fundamental aspect of game theory lies in the Nash solution concept which, however, requests stronger assumptions on agents' information (type, strategy, utility, etc.). There are some related solution concepts, such as dominant strategy and Bayesian-Nash strategy in game theory. In this paper, we initially attempt to implement Nash solution concept in evolutionary computation (EC) in the context of a mechanism design problem.

The history of mechanism design can be traced back to the 1920s to the 1930s, when there was an economic controversy concerning the socialist economic system. Liberal economists, such as von Mises and von Hayek, believed that socialist economics cannot obtain effective information to make their economic system operate efficiently [3, 4]. However, other economists, such as Lange, thought socialist economics can solve the problem of requesting more information about economic operation so as to promote efficient resource allocation [5]. The contention of this controversy focuses on the information issue, which is also a core problem of mechanism design. Hurwicz established the fundamentals of mechanism design theory from the information viewpoint, which proposed a common framework to compare the issue of efficiency among different economic systems [6].

Mechanism design theory can be considered as a comprehensive utilization of game theory and social choice theory, which is referred to as principal agent theory and implementation theory as well. Its primary philosophy is to design a series of rules to implement the trust between principal and agent and to ensure the mechanism runs well under an asymmetric information condition. The fundamental issues of mechanism design refer to (1) whether there is a set of rules in a game and (2) how to implement these rules. The objective of mechanism design is to achieve a preset objective that a game establishes, when all agents act for their own benefit with their private information.

When we make reference to individuals in EC, they are neither rational nor self-interested participants in an EC algorithm. The individuals just follow the fixed rules of the EC algorithm. In this paper, we assume that they are rational, self-interested, and more “intelligent” agents in an EC algorithm, so that we can handle the design of EC algorithm as a mechanism design problem. The design principle of an EC algorithm should be more reasonable based on this assumption, rather than merely simulating natural phenomenon in an iterative process of evolution. There are several issues that need to be discussed in this context: (1) what kind of information should be involved in mechanism design of EC algorithm; (2) how to distinguish principal(s) and agent(s) in a game of EC algorithm design; (3) which solution concept should be implemented in this game; (4) how to define the expected utility function for an established mechanism or model; (5) whether the established mechanism or model is the optimal one; and (6) what are the characteristics and properties of designed game induced by EC. After modelling EC as a game, we can introduce theoretical principles of game theory and microeconomic theory into the fundamentals of EC for designing, enhancing, and improving more efficient and effective EC algorithm. This is the primary motivation and original contribution of this work.

Following this introductory section, in Section 2, we make a brief review of some basic definitions, notations, and related works on game theory, mechanism design, and EC. The primary concepts and works include mechanism design, Nash equilibrium, EC, and interdisciplinarity of these aspects. We establish some basic concepts on algorithmic mechanism design of the EC and introduce a formal framework that implements Nash equilibrium into EC for solving strategy selection issue in Section 3. The formal framework can be implemented in any EC algorithm by designing agent, utility, strategy, and equilibrium solution (such as Nash equilibrium, Baysian-Nash equilibrium, and dominant equilibrium) in corresponding EC algorithm in detail. In Section 4, we design the proposal in a differential evolution algorithm and evaluate its performance by designing a series of comparison experiments. Based on the evaluation results, we make a comprehensive discussion on algorithmic mechanism design of the EC, Nash equilibrium implementation in EC, and so forth, in Section 5. Finally, we present some future works and open topics towards the final objective of our proposal, that is, an algorithmic mechanism design of EC, in Section 6.

## 2. Definitions, Notations, and Related Works

### 2.1. Mechanism Design Problem

The mechanism design problem can be described as follows. There is a system with *I* agents (*i* = 1,2,…, *I*) and a set of outcomes (denoted as *O*). Each agent has private information about its utility corresponding to the outcome. Other information in this system is public knowledge or public information. The private information is the agent's type (denoted as *θ*_*i*_ ∈ Θ_*i*_) that determines its utility (*o*_*i*_ = *u*_*i*_(*θ*_*i*_)) among different outcomes (*o*_*i*_ ∈ *O*). The set of type (Θ_*i*_) presents all the possible information available to the agent *i*. The mechanism design problem is to solve an optimal solution (*o* ^*∗*^ = *f*(*θ*), *θ* = (*θ*_1_, *θ*_2_,…, *θ*_*I*_)) of an implementation problem given by (1) with agents that have private information about their actions. Equation (1) is a social choice function, which generally selects an outcome with maximum total utility in the system (2) [8]. Consider

(1) f:Θ1,Θ2,…,ΘI⟶O,

(2) fΘ=arg⁡max⁡∑i=1Iuiθi.

Mechanism presents a set of strategies (*s*_*i*_(*θ*_*i*_)) of each agent and methods used to select an outcome based on the strategies. Game theory is used for analysing the outcome of a mechanism. On the contrary, the mechanism design is used for implementing a social choice function (1), if the outcome can be obtained by an (some) equilibrium strategy(ies). Finding an equilibrium strategy is the final objective of mechanism design. The equilibrium strategy can be Nash equilibrium, Bayesian-Nash equilibrium, dominant equilibrium, or other solution concepts. The better solution of a mechanism design is as strong as possible.

### 2.2. Nash Equilibrium

Nash equilibrium (NE) is a well-known solution concept in game theory, which computes the outcome of a game with self-interested agents based on certain assumptions on information available about each other [2]. It states that every agent selects a strategy with maximizing utility in equilibrium by considering every other agent's strategies. Suppose that *s* = (*s*_1_, *s*_2_,…, *s*_*I*_) is a joint strategy of all the agents (there are *I* agents) and *s*_−*i*_ = (*s*_1_, *s*_2_,…, *s*_*i*−1_, *s*_*i*+1_,…, *s*_*I*_) is a joint strategy of all the agents except agent *i*. A Nash equilibrium is a strategy profile *s* = (*s*_1_, *s*_2_,…, *s*_*I*_), if every agent maximizes its utility. It is shown in (3), where *s*_*i*_′ ≠ *s*_*i*_. Note that *s*_*i*_(*θ*_*i*_) and *s*_−*i*_(*θ*_−*i*_) present the strategy of agent *i* under its type *θ*_*i*_ and strategies of other agents without agent *i* under their types *θ*_−*i*_, respectively:

(3) $$\begin{array}{c} u_{i}\left(\;s_{i}\left(\;\theta_{i}\;\right),s_{-i}\left(\;\theta_{-i}\;\right),\theta_{i}\;\right)\geq u_{i}\left(\;s_{i}^{\prime }\left(\;\theta_{i}\;\right),s_{-i}\left(\;\theta_{-i}\;\right),\theta_{i}\;\right). \end{array}$$

A strategy profile is in Nash equilibrium that means every agent can obtain maximum utility with strategy *s*_*i*_. The disadvantage of Nash equilibrium is that it makes very strong assumptions about agent types and information about other agents. It must have relative enough information about preferences of every agent and agents with common knowledge to play a Nash equilibrium in a one-shot game. There is as well an important issue that all agents must select the same Nash equilibrium.

### 2.3. Evolutionary Computation

Evolutionary computation (EC) is a series of stochastic optimization algorithms that are inspired originally by natural selection and “survival of the fittest” and further developed by ant colony optimization, artificial immune systems, partial swarm intelligence, and others. From the algorithmic taxonomy viewpoint, EC takes the probability theory as its philosophy and methodology. The fundamentals of its search mechanism are established in the basics of probability theory. The system behaviour of an EC algorithm can therefore be presented as a probability transition matrix, and its dynamic optimization process can be described as a Markov chain [9]. However, other theoretical analysis methods from deterministic theory, such as fixed point theory, are also introduced into the EC in order to study its fundamental aspects [10], such as efficiency, effectiveness, and convergence. Drawing inspiration from chaos theory and its ergodicity, a chaotic evolution algorithm has been recently proposed and studied [11]. This is very different from the conventional deterministic and stochastic optimization algorithms. Chaotic evolution can be considered as an implementation of the chaotic optimization algorithm, whose theoretical fundamental is supported by chaotic philosophy and methodology.

There are two components in EC from the viewpoint of the algorithm design framework. One is an iterative process, and the other is one or several evolutionary operations that are implemented by a variety of methods. Algorithm selection and parameter settings are two critical issues, when we apply EC to an optimization problem. The objective of the former issue is to answer the question as to which is the best EC algorithm to solve a concrete problem. The latter one seeks to obtain the best parameter setting of an EC algorithm to obtain a better optimization performance. This paper attempts to solve and answer the latter problem from the viewpoint of algorithmic mechanism design and tries to find the fundamental aspects of EC from this perspective.

### 2.4. Differential Evolution

Differential evolution (DE) is a population-based optimization algorithm, as well as a member of an EC family [12]. DE uses a differential vector from two random individuals to perturb a base vector (a third random vector from a population) to implement a mutation operation and obtain a mutant vector. It conducts a crossover operation between the mutant vector and proceeded target vector to create the trial vector. Following this, it compares the fitness of the target vector and trial vector to allow the better one to survive into the next generation. The formal expression of this search mechanism is shown in (4), where mutant_*i*,*j*_ is a mutant vector, base_*i*,*j*_ is a base vector, and *x*1_*i*,*j*_ and *x*2_*i*,*j*_ are two random vectors (*i* and *j* are the indexes of individual and dimension, resp.). *F* is a scale factor that needs to be set. Note that the target vector, base vector, and two random vectors are four different vectors, so the minimum population size is four in DE. The pseudocode of DE is shown in Algorithm 1. Consider

(4) $$\begin{array}{c} {\text{mutant}}_{i,j}={\text{base}}_{i,j}+F*\left(\;x1_{i,j}-x2_{i,j}\;\right). \end{array}$$

For improving DE performance, a self-adapting control parameter method is proposed in DE (jDE) to implement a variety of selections of DE parameters [7]. The crossover rate (Cr) and scale factor (*F*) are encoded in each individual in jDE, which are adjusted by (5) and (6) every generation, respectively. The parameters, *τ*_1_ and *τ*_2_, are both set to 0.1, and rand⁡[*x*, *y*] is a random value from a uniform distribution within (0,1]. From (5) and (6), *F*_*i*_ and Cr_*i*_ are updated in a small probability:

(5) Fi=rand⁡0.1,1if rand⁡0,1<τ1Fiotherwise,

(6) Cri=rand⁡0,1if rand⁡0,1<τ2Criotherwise.

### 2.5. Evolutionary Computation Meets Game Theory and Mechanism Design

Game theory attempts to determine the outcome of a game with a set of given strategies from self-interested agents, and mechanism design seeks to design the strategies of agents to obtain the desired outcome in a game. The research objectives of game theory and mechanism design are to find outcomes corresponding to strategies and to design strategies under a desired outcome, respectively. However, EC tries to find the optimal solution(s) of an optimization problem. These three disciplines, game theory, mechanism design, and EC, are quite different with regard to research philosophies, approaches, or objectives.

Game theory and mechanism design have been introduced into some fields, such as distributed artificial intelligence [13], resource allocation [14], and scheduling [15]. It is easy to establish concrete models of agent and utility function by using game theory in corresponding applications. Mechanism design was also reported in computation related topics [16] and algorithmic design problems [17], which presents complexity bounds and worst case approximation. These studies focus definitely on the same viewpoint, that is, a mechanism design problem of deterministic optimization. These works do not relate to EC and do not pursue our proposal, that is, an algorithmic mechanism design of the EC, which is a stochastic optimization method rather than deterministic optimization one.

From the related literatures and to the best of our knowledge, EC can act as an optimization tool to find the best response, equilibrium of strategy in game theory and mechanism design. Some EC algorithms, such as genetic algorithm [18], genetic programming [19], and coevolution [20], are applied to game theory problems to obtain the best strategy or parameters. Scant literature reports having applied the philosophy and methodology of game theory or mechanism design to the fundamental aspects of EC. This paper attempts to conduct some initial work in this area.

## 3. Algorithmic Mechanism Design of Evolutionary Computation: A Strategy Equilibrium Implementation Problem

### 3.1. Motivation of the Proposal

Conventional EC algorithm as a search method is applied to an optimization problem with a set of fixed algorithm parameters and operations. Although the inspiration of EC seeks to find adaptive mechanisms in its search scheme, the fixed parameter setting restricts its optimization capability. From the system theory viewpoint, the whole EC algorithm system can be considered as a control system and its parameters decide the system behaviour. If we aim to obtain the best optimization performance, the parameters of EC algorithm should be controllable, and the relationship between parameter settings and optimization performance should be observable. However, because the EC algorithm belongs to stochastic method, such deterministic methods (e.g., automatic control method) have not been applied to the EC area in order to study on its theoretical fundamentals.

There are primary three research directions for improving optimization performance of an EC algorithm [21]. The first is obtaining information from a fitness landscape, such as fitness landscape approximation, and using the information to conduct a special operation or to develop new search schemes for tuning the parameter of an EC algorithm [22]. The second is developing new mechanisms in extant EC algorithm to enhance its performance or to implement the parameter adaptive mechanism [23]. The jDE algorithm that is introduced in Section 2.4 belongs to this aspect. The third direction is creating new EC algorithms or metaheuristics with less parameters and more natural adaptiveness in order to enhance EC algorithm [11].

Individuals in conventional EC algorithms or in some population-based optimization algorithm are common elements, which present the search space and structure aspect of an optimized problem. Individuals search for the optimum/optima with the information shared between one another, so they are influenced each by the other from one generation to the next. They operate under the same evolutionary operations with fixed operation rates from parameter settings in a certain EC algorithm. This work scheme restricts the EC algorithm search capability.

If we consider individuals in EC algorithm as agents, the EC algorithm therefore can be considered as a game, whose outcome is optimal solution(s) or some other metric(s). Furthermore, these agents play the game (i.e., EC algorithm) with self-interested preference and conduct optimization strategies with their own preferences. An EC algorithm designer can play this game by using noncooperative or cooperative game concepts. The objective of the EC algorithm design is to find optimum/optima by designing a proper strategy for these individuals (i.e., agents). This description can be abstracted as a mechanism design problem. That is, design, parameter setting, and operation selection of the EC algorithm can be decided by the individuals, and the desired outcome is to find the final optimum/optima. EC algorithm can be modelled as a game, that is, an agent system, so the corresponding theoretical fundamentals of agent system, game theory, or mechanism design can be brought to bear on the study of the fundamental theoretical aspects of EC algorithm. This philosophy and motivation highlight the originality of this paper.

### 3.2. Strategy Equilibrium Implementation in Evolutionary Computation Algorithm

We propose that the design of an EC algorithm can be considered as a mechanism design problem. In this section, we briefly introduce some concepts in game theory and their corresponding explanations and implementations in EC. Based on these concepts, we establish a formal EC algorithm framework by using the equilibrium concept and solve this mechanism design problem by finding the Nash strategy equilibrium in EC algorithm.

#### 3.2.1. Agent and Its Type

An agent is an abstract concept in game theory. It refers to a participant in a game who will make strategic decisions based on its type. Type (Θ) of an agent determines preference of the agent by considering all the outcomes of a game, as it is mentioned in (1), (2), and (3). As usual, *θ* ∈ Θ refers to the type of an agent in game theory.

Individuals in EC are definitely considered as agents under the basic philosophy of our proposal. In game theory, an agent is treated as being self-interested. However, in EC, we consider it as a more rational one that allows itself to select certain operations, even though its utility will become low. For example, the simulated annealing mechanism in EC is such a case, if the EC algorithm allows individuals to be replaced by their offspring with worse fitness. The agent in a game can determine its own behaviour, so the individuals of EC should follow this rule by encoding operation types and their rates in themselves. In the EC, the type of an agent can be considered as information, such as fitness and fitness landscape, or some metrics of the evolution.

#### 3.2.2. Strategy

Strategy concept in game theory presents a set of actions, or decision rules, which define the action of an agent. It is decided by the agent type (*θ*), and *s*_*i*_(*θ*_*i*_) presents agent *i* selects strategy *s*_*i*_ due to its type *θ*_*i*_. The strategy of an agent can be deterministic or stochastic. In game theory, the deterministic strategy and stochastic strategy are referred to as pure strategy and mixed strategy, respectively. A strategy is a function of the agent type, as well as being an expression of the utility of the agent. They are interdependent concepts, whereby the utility of an agent is a function of the strategy. EC operators and parameters and their selection issues can be considered as the strategy and strategy selection problem in EC algorithm design. They can be decided by each individual according to their own type, that is, the information obtained in the evolutionary search process.

#### 3.2.3. Utility

The utility of an agent is a function that decides preference of agent under all the outcomes (*O*) and types (Θ). An agent prefers outcome *o*_*a*_ to outcome *o*_*b*_, when *o*_*a*_ > *o*_*b*_ (note that *o* = *u*(*θ*) and *θ* is a parameter), so the measurement of utility is used to decide agent strategy. The utility concept in EC refers to some measurements of algorithm optimization performance, such as fitness improvement, evolution successful rate, or other metrics that can evaluate algorithm performance or individual performance.

When we describe a mechanism design problem, two issues should be stated clearly. One is the domain of agent preference that determines what an agent refers to, and the other is solution concept, that is, what the outcome specification is. In this paper, we primarily discuss the second issue, that is, implementation of the solution concept of an EC algorithmic mechanism design, and simplify that the agent (individual) has a quasilinear preference. In (7), *v*_*i*_(*x*, *θ*_*i*_) is valuation function, *x* is a choice of agent, and *p*_*i*_ is payment by agent. In our evaluation section, we use fitness improvement (8) as the utility (it presents valuation and payment of an agent as well) to determine an individual's strategy:

(7) $$\begin{array}{c} u_{i}\left(\;o,\theta_{i}\;\right)=v_{i}\left(\;x,\theta_{i}\;\right)⟺p_{i}. \end{array}$$

#### 3.2.4. Strategy Equilibrium

The agent in EC algorithm is the individual, which is a participant in a game of EC. Each individual can decide their own strategy, that is, operation and operation rate, by their utilities and types, which can be measured as fitness improvement information or other algorithm evaluation metrics. All the EC algorithm implementations can be abstracted as a mechanism design problem, whose equilibrium concept is a solution of the problem. As all types of equilibrium concept can be a solution of our established framework, a Nash equilibrium is a related easy and weak solution in a game because of Dominant≻Baysian_Nash≻Nash. The objective of mechanism design is to implement some strategy equilibrium concepts in a game; however, it is an optimization problem, in which equilibrium implementation can lead to the best performance and fast convergence of EC algorithms. We initially discuss, design, and evaluate our proposed framework by implementing Nash equilibrium, but it is not limited to within Nash equilibrium.

#### 3.2.5. Mechanism Design of Evolutionary Computation Algorithm

We define and describe EC algorithm as a mechanism implementation problem. Given an EC algorithm EC = (Σ_1_, Σ_2_,…, Σ_*n*_; *g*(·)), it defines a strategy set Σ_*i*_ to each individual and an outcome rule *g* : Σ_1_ × Σ_2_ × ⋯×Σ_*n*_ → *O* that is the outcome implemented by the mechanism (EC algorithm) for strategy profile *s* = (*s*_1_, *s*_2_,…, *s*_*n*_). EC algorithm (a mechanism, EC = (Σ_1_, Σ_2_,…, Σ_*n*_; *g*(·))) implements social choice function *f*(*θ*) (1), if *g*(*s*_1_^*∗*^(*θ*_1_), *s*_2_^*∗*^(*θ*_2_),…, *s*_*n*_^*∗*^(*θ*_*n*_)) = *f*(*θ*) and *θ*_*i*_ ∈ Θ_*i*_. Strategy (*s*_1_^*∗*^, *s*_2_^*∗*^,…, *s*_*n*_^*∗*^) is an equilibrium solution to the game induced by EC.

There are some different aspects in this description of EC, when we consider it as a mechanism implementation problem. First, individuals of EC algorithm can select their own strategy depending on their types, that is, the information they obtain, rather than process a fixed EC operation in conventional EC. Second, we should define type, strategy, and utility of individual to describe the game induced by EC. Third, we should compute some forms of equilibrium solution for an EC algorithm implementation in every generation or several generations (gen) once for achieving best optimization performance of EC. Fourth, the domain of agent preference should be defined reasonably to achieve the correct decision-making of strategy selection. From this description of EC by considering it as an algorithm mechanism design problem, EC algorithm is a set of algorithm strategies and outcome rules. The optimization performance of EC algorithm is decided by strategy and strategy selection. The study of EC convergence or performance improvement is transferred to the study of the relationship between strategy selection (equilibrium implementation) and EC algorithm performance. It provides us with an opportunity to discuss the theoretical aspects of EC within this framework.

### 3.3. A Case Study: Nash Strategy Equilibrium-Based Differential Evolution Algorithm

After we introduce the optimization process of EC as an algorithmic mechanism design problem, there are a variety of ways to implement EC algorithms by designing concrete implementations of a game. In this section, we design a concrete implementation of an EC algorithm by considering Nash strategy equilibrium and take DE algorithm as a case to study how to design our proposal in an EC algorithm. There are two design issues that should be concentrated on especially. One is the definition of strategy; the other is equilibrium calculation. In Nash strategy equilibrium-based DE, operations and their parameters are coded with each individual, so that the individual can select their own operation and rate. Mutation method, crossover rate, and scale factor rate are three primary parameter settings in DE. For simplifying the design objectives, we design the algorithm by splitting individuals into two groups (Group A and Group B) with equal population size and strategy sets within the following criteria:

- Mutation = {DE/best/1/bin, DE/rand/1/bin}.
- Crossover rate (Cr) = {1 ≥ Cr > 0.5, 0 < Cr ≤ 0.5}.
- Scale factor (*F*) = {1 ≥ *F* > 0.5, 0 < *F* ≤ 0.5}.

There are two participants (Group A and Group B) who join in the designed DE algorithm. Every individual in the designed DE algorithm can select a mutation method from either “DE/best/1/bin” or “DE/rand/1/bin,” crossover rate, and scale factor either more than 0.5 or less than 0.5. We define the utility (payoff) of agent (individual group) as the fitness improvement (8), where fitness(*∗*)_*n*_ and fitness(*∗*)_*n*−1_ are fitness of individual *∗* in *n* and *n* − 1 generations. For example, for a minimum optimization problem, the less a fitness improvement value the better, but our proposed framework is not limited to within this definition of utility. After one generation or several generations (gen in Algorithm 2), we calculate the fitness improvement of every individual and sum total fitness improvements of Group A and Group B with different strategies and obtain the payoff matrix of different strategy selection combinations (Table 1). We have

(8) $$\begin{array}{c} \text{Fitness}\;\text{Improvement}=\text{fitness}{\left(\;*\;\right)}_{n}-\text{fitness}{\left(\;*\;\right)}_{n-1}. \end{array}$$

With the payoff matrix and the definition of Nash equilibrium as in (3), we can find that the strategy (*s*_*a*_^*∗*^, *s*_*b*_^*∗*^), which satisfies ((Pay_*a*,*s*_*a*_^*∗*^_, Pay_*b*,*s*_*b*_^*∗*^_)⪰(Pay_*a*,*s*_*i*__, Pay_*b*,*s*_*i*__)), is a Nash strategy equilibrium in our designed DE. From this definition, we can find that (Pay_*a*,*s*_*a*_^*∗*^_, Pay_*b*,*s*_*b*_^*∗*^_) is a dominant solution of the game as well. In our established EC framework, a Nash equilibrium solution can be a Pareto solution and dominant solution. If Nash equilibrium solution is a Pareto solution, it presents maximal optimization capability of the algorithm that decides the convergence property of the algorithm. This is a critical property of our proposal. In the next generation, if the individual strategy is the Nash equilibrium strategy, they keep their strategies as they are. Otherwise, an individual will tune its strategy with a random guess. By considering these design elements into a canonical DE, Nash strategy equilibrium-based DE algorithm is shown in Algorithm 2.

## 4. Numerical Evaluations

### 4.1. Evaluation Design

The objective of our proposal is to design an EC algorithm from the viewpoint of game theory and mechanism design. After we find the Nash equilibrium strategy, the EC algorithm should be enhanced practically. In the fundamental aspects of EC algorithm, approaches and theories of EC will be therefore explained by the corresponding context of game theory and mechanism design. In this section, we design a series of evaluations to verify the effectiveness and efficiency of our proposal.

We use 14 benchmark functions from [24] in evaluation.  Table 2 shows the type, characteristic, bound, and optimum of these benchmark tasks. They include landscape characteristics of being shifted, rotated, global on bounds, unimodal, and multimodal. We test them with three types of dimension setting, *D* = 5 (5 dimensions (5D)), *D* = 10 (10D), and *D* = 30 (30D).

### 4.2. Evaluation Setting

We compare our proposed Nash strategy equilibrium- (NE-) based DE algorithm (i.e., best-*F*, rand-*F*, best-Cr, rand-Cr, mutation, and mixture) with canonical DE and jDE.  Table 3 presents the abbreviations of algorithms and their parameter settings, scale factor (*F*), and crossover rate (Cr) of two canonical DE (DE-best and DE-rand) are both set at 1 from the discussion of [12]. We test each function with 30 independent runs and 1000 generations for each run. Every benchmark function is a minimum optimization problem, that is, the smaller fitness value means the better optimization performance.

Because the production of differential vectors in DE is a critical factor that influences optimization performance and one of our evaluation objectives is to investigate the strategy selection influence of the DE algorithm, we need to reduce the diversity of differential vectors in the evolution so as to reduce population diversity influence and to enhance strategy selection influence. As the minimum number of a DE population size is four (PS = 4), we therefore use it in our evaluation to reduce the diversity influence as much as possible. For a fair comparison of these algorithms with normal conditions, we also set the population at PS = 10*∗D* to check their performance from discussion of [12]; that is, PS = 50, PS = 100, and PS = 300 for *D* = 5, *D* = 10, and *D* = 30, respectively.

In NE-based DE algorithm, there is a parameter (gen in Algorithm 2) to decide in which generation an NE strategy is calculated. In our evaluation, we set gen = 1 that means the NE strategy is calculated every generation. If individuals have these NE strategies, they keep them; otherwise, they select a strategy from a strategy pool with a random guess.

### 4.3. Evaluation Metrics

Several evaluation metrics are involved in the analysis and discussion. We apply Wilcoxon signed-rank test and Friedman test on the fitness value at 1000th generation to check the significance of the proposal and make an algorithm rank. Tables 4, 5, and 6 are the mean values of each benchmark function with 5D, 10D, and 30D, respectively. Wilcoxon signed-rank tests are applied between comparison algorithm and our proposed NE-based DE algorithms.

**Figure 1:**
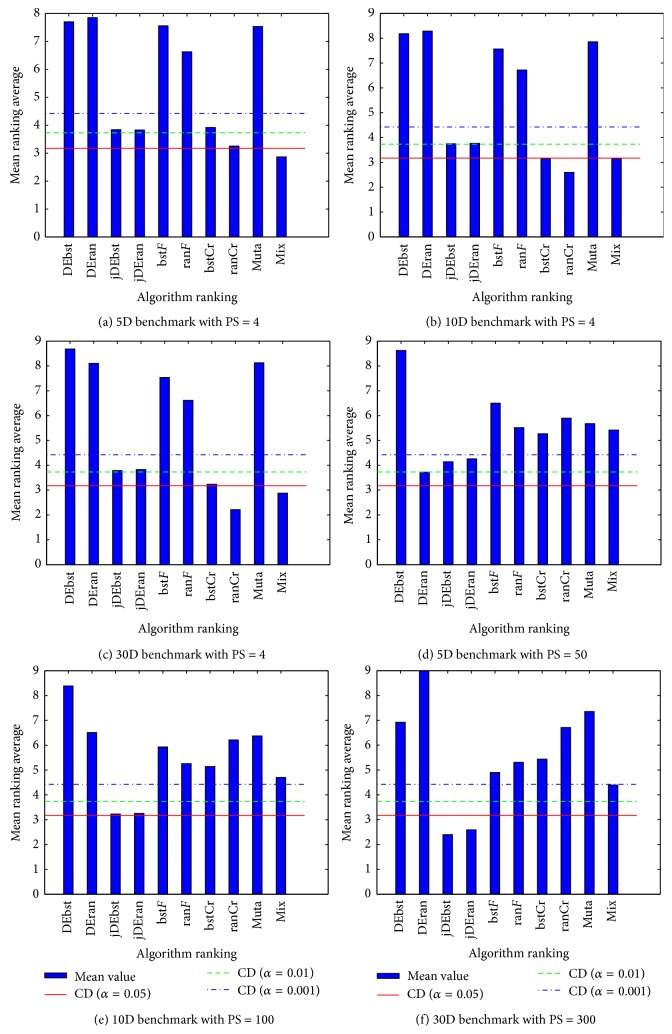
Bonferroni-Dunn test of 10 algorithms and 14 benchmark functions with 5D, 10D, and 30D in significant levels of *α* < 0.001, *α* < 0.01, and *α* < 0.05; each group takes the minimal rank algorithm as a control algorithm. DEbst, DEran, jDEbst, jDEran, bst*F*, ran*F*, bstCr, ranCr, muta, and mix present the short forms of algorithms in Table 3. We can observe that our proposed NE-based DE can significantly win in PS = 4 groups, the same as DE and jDE in some significant levels in PS = 10*∗*Dimension groups, except 30D benchmark function with PS = 300.

After obtaining the average mean rank of each algorithm from Friedman tests, we apply Bonferroni-Dunn tests in significant level of *α* < 0.001, *α* < 0.01, and *α* < 0.05 on 5D, 10D, and 30D benchmark problem. Critical difference (CD) used in Bonferroni-Dunn test is in (9), and *k* = 10 and *N* = 14, *q* is equal to *q*_*α*_(0.001) = 3.865, *q*_*α*_(0.01) = 3.261, and *q*_*α*_(0.05) = 2.773 from Appendix Table B.16 of [25].  Figure 1 demonstrates the visual presentations of critical differences among these algorithm ranks. We have

(9) $$\begin{array}{c} \text{CD}=q_{\alpha }\sqrt{\frac{k\left(k+1\right)}{6*N}}. \end{array}$$

Number of function calls (NFC in (11)) before the desired reached fitness value (VTR in (10)), accelerate rate (AR in (13)), and success rate (SR in (12)) are introduced as well to evaluate our proposal. Tables 7 and 8 present these results. Consider

(10) VTR=average fitness of each method at MAXNFCth generation,

(11) NFC=average # of fitness calculations until convergence reaches VTR,

(12) SR=# of reaching to VTR# of generations,

(13) AR=NFCordinalDENFCproposal.

## 5. Analyses and Discussions

### 5.1. Philosophy of the Proposal

A good researcher should think outside the conventional philosophy and methodology of a particular field and consider a broad view rather than focus on small matters. The objective of this study is not only to find a method for designing, enhancing, and accelerating EC from the viewpoint of an algorithmic mechanism design problem, but also to establish EC fundamental aspects by borrowing from game theory and mechanism design.

There are three parallel ways to research and consider our world from the philosophies of determinism, probability, and chaos. In the optimization field, there are also three categories of optimization method from the corresponding philosophy and methodology, that is, deterministic, stochastic, and chaotic optimization methods. EC belongs to the stochastic one, and its fundamental aspect should be described from the probability viewpoint. This restricts the fundamental development of EC and explanation capability of its algorithms. This study tries to use fundamentals from game theory and mechanism design (deterministic theory) to explain EC (stochastic algorithm) and establish its fundamental contents. This is one of the contributions of this paper.

From the description of EC in Section 3.2.5, EC is a mechanism that is composed of strategy and outcome rules. Different equilibrium decides the optimization performance of an EC algorithm. EC convergence lies in the effective strategy equilibrium design and implementation. This is another contribution made by this paper. This provides another view for the understanding of EC algorithms from the fundamentals of game theory and algorithmic mechanism design.

### 5.2. Performance of the Proposal

From the observations of mean values in Tables 4, 5, and 6, our proposal can obtain a significantly accelerated convergence in benchmark functions. In Table 4, most of the proposals outperform DE-best and DE-rand, and some of them outperform jDE-best and jDE-rand. Optimization performance applied to multimodal tasks by means of our proposals is better than that applied to unimodal tasks. The same observations can also be made with regard to the 10D and 30D benchmark tasks in Tables 5 and 6. This may be because the capability of escaping the local optimum by means of our proposal is better than that of DE and jDE. However, the deep exploitation capability of our proposal is weak. This indicates that the strategy selection of our proposed EC algorithm has both high exploration capability and weak exploitation capability. It needs to be further investigated and proved by both empirical and theoretical studies in our future work.

Algorithms with NE strategy scale factor are more effectively applied to multimodal 5D benchmark tasks than to unimodal ones by comparing best-*F* and rand-*F* algorithms applied to the *F*1–*F*5 group and *F*6–*F*14 group in Table 4. The same conclusion cannot be found in 10D and 30D benchmark tasks in Tables 5 and 6. It seems that the population diversity caused by scale factor is more efficient for the improvement of optimization performance in high dimension tasks than that in low dimension tasks. Algorithms with NE strategy scale factor have lower acceleration performance than those with NE strategy crossover. This can be explained from the viewpoint of population production diversity by these two mechanism implementations.

Algorithms with NE strategy crossover are more effective in all dimension settings, that is, 5D, 10D, and 30D benchmark functions. When the complexity of a task increases, the effectiveness appears more explicitly. This can be observed by comparing Wilcoxon signed-rank test results between the two algorithms (best-Cr and rand-Cr) in 5D, 10D, and 30D benchmark tasks in Tables 4, 5, and 6, respectively. The effectiveness and efficiency of our proposal in high dimension and complex benchmark problems are demonstrated in this way.

In Tables 4, 5, and 6, only a few benchmark tasks are accelerated by our proposal with NE strategy mutation, where an individual selects either the best vector or a random vector as the best vector. This indicates that individual searching in the different region (around the best individual or a random individual) is not a serious factor of performance influence in DE with our proposed NE strategy.

### 5.3. Nash Equilibrium Implementation Inclusion

Mixture algorithms include three NE strategies, that is, NE strategy of *F*, NE strategy of Cr, and NE strategy of mutation. It can obtain the best optimization performance from these algorithms with a single NE strategy because of implementation inclusion. The evaluation results and Wilcoxon signed-rank tests from Tables 4, 5, and 6 do prove this conclusion. However, the performance of algorithms with mixture NE strategies is not completely equal to algorithms with single NE strategies. For example, best-Cr and rand-Cr are two algorithms with more winner cases from Wilcoxon signed-rank test, but this is not the same as the winner cases of mixture algorithms in Tables 4, 5, and 6.

From the NFC, SR, and AR metrics (Tables 7, 8, and 9), rand-Cr algorithm is the one with the least NFC and greatest SR, and jDE-best and mixture algorithms have the first and second winners in AR metrics in all dimensional setting benchmark tasks, respectively. These observations also indicate that DE with our proposed NE strategy can achieve the desired fitness improvement target with the less computational cost. Although the mixture algorithm is not the winner, there are not many differences between AR values of jDE-best and mixture in all dimensional setting benchmark functions. From a statistical viewpoint, they are not significantly different. EC with pure strategy (canonical DE) has less optimization performance than that obtained by EC with mixed strategy (proposals) from our empirical evaluation. Although some other researches have the same empirical evaluation result, they do not all explain the reason for EC algorithm improvement from the viewpoint of game and mechanism design.

### 5.4. Algorithm Rank

We apply Friedman test on the 10 algorithms in our evaluation. The algorithms, best-Cr and mixture, win the most of first ranks in 5D, 10D, and 30D benchmark tasks when PS = 4. We can conclude that best-Cr is the winner for unimodal benchmark task, and mixture is the winner for the multimodal benchmark task. It is the same observations from Wilcoxon signed-rank test of mean values. However, for the population setting as PS = 10*∗*Dimension groups, best-jDE and rand-jDE are the winners. It is primarily due to the population diversity increasing the optimization capability of DE algorithm, whose optimization capability is decided by the differential vector production.

In evaluating the critical difference of mean rank scores of each algorithm, we apply an additional Bonferroni-Dunn test on the results of the Friedman tests (Figure 1). In each comparison group, we take the algorithm with the least ranking as a control algorithm. We can make the conclusions that our proposed method has a significant difference with canonical DE (even with jDE in some significant levels). And the mixture method, rand-Cr method, and rand-Cr method are the winner ones for 5D, 10D, and 30D benchmark tasks in PS = 4 setting groups, respectively. Except 30D benchmark functions with PS = 300 setting, there is not any significant difference in PS = 10*∗*Dimension setting groups with the significant level of *α* < 0.05. The exception case indicates that Nash strategy equilibrium implementation is not the best solution concept in our case study, that is, DE algorithm.

### 5.5. Nash Equilibrium and Mechanism Design of Evolutionary Computation

By considering EC optimization as an algorithmic mechanism design problem, we find a way to describe EC and establish its formal framework. There are several issues that need to be further discussed and investigated.

#### 5.5.1. Domain of Agent Preference

Information is critical element in a game and mechanism design problem. It decides the next response that will be played by agents. Fitness, fitness landscape, and other preset algorithm evaluation metrics can be involved as information in mechanism design of EC algorithm. There are some principles that should be followed when introducing information for each individual. The first is that the information is easy to be obtained with less computational cost, that is, easily retrievable. The second is that the information can support useful element or factor for individual to operate correct action. The third is that the information should be explainable to individual between the action and corresponding information.

For mechanism design in conventional economic field, known as principal and agent theory, it distinguishes different roles, such as principal and agent, in its framework. Agent in game and mechanism design should be rational and self-interested. However, in mechanism design of EC algorithm, it does not need to distinguish these different roles. We consider these individuals as equal participants in the EC, but they should follow principles of rationality and self-interest. It means there must be some reasonable relationship between their action and the information they obtain.

Domain of agent preference is one of the properties of mechanism. In this paper, we try to implement an NE strategy in designed EC algorithm but ignore the domain of agent preference issue that defines choice and payment of an agent. We simplify the agent with an assumption of quasilinear preference (7). We will continue to discuss and investigate other domains of agent preference in EC algorithm design.

In mechanism design of EC algorithm, the information of individual can be the same as expected payoff function. So it is easy to reduce design element when we implement a mechanism design of EC algorithm. We can use the fitness improvement information as the payoff (utility) of each agent in our NE strategy-based DE in this paper. However, payoff concept supports us with more possibility to implement different mechanism design. We will consider this design element in our future work to investigate the algorithmic mechanism design and implementation of EC algorithm.

#### 5.5.2. Solution Concept

The solution concept is important to the mechanism implementation of a game. As we described in Section 3, the objective of the mechanism design of EC algorithm is to design a set of possible strategies and outcome rules for implementing a social choice function in the form of a solution concept. Some solution concepts, such as Nash equilibrium, Bayesian-Nash equilibrium, and dominant equilibrium, can be concrete implementations. Definitely, the stronger the solution concept, the better, because it makes less assumption on an agent. In this study, we take DE as an example to implement an EC algorithm mechanism design by using the weak solution concept (i.e., Nash equilibrium). We will investigate the possibility of other solution concepts' implementation and the relationship between equilibrium solution implementation and EC optimization performance in the future.

In our mechanism design for an EC algorithm, we found an interesting property in that the NE solution is a dominant solution in established mechanism design. Because the influence of each individual's improvement is decided by their position and the differential vectors they made in past generation, the fitness improvement information (equal to payoff) of one group is a constant value for all the strategies of the other groups. So the payoff matrix can be expressed as in Table 1.

We prove this conclusion with a case study from the viewpoint of combinatorial theory. Suppose that there is a game on mechanism design of EC algorithm with *i* individuals, and each individual has *s*_*i*_ strategies (*i* = 1,2,…, *N*). The total number of payoff combinations is given by (14). However, in our designed mechanism design of EC algorithm, the strategy of every individual has only one payoff whatever other individuals play strategies. The total number of payoff combinations of our proposal is given by (15). This means every possible combination of payoff can appear, so within this there must be found the combination with all the elements that is the maximum value, that is, a Nash equilibrium solution, as well as a dominant solution. We have

(14) payoff=∑si=1N∑k=1si∏j=1,j≠iNsj,

(15) payoff=∏j=1Nsj.

### 5.6. Pareto Optimal Solution and Evolutionary Computation Convergence

In game theory, mechanism implements a social choice function, and strategy profile is an equilibrium solution to the game induced by the mechanism. In EC, if we implement a strategy profile following certain equilibrium solutions to achieve Pareto optimal solution, it means the optimization capability of the EC algorithm achieves its maximum state and cannot obtain further better results any more. In this sense, EC convergence is related with this state; that is, the strategy equilibrium solution of EC implements a Pareto optimal solution. We can discuss the EC convergence issue under this assumption.

Social choice function *f*(*θ*) is the Pareto optimal if for every *o*′ ≠ *f*(*θ*) and all types *θ* = (*θ*_1_, *θ*_2_,…, *θ*_*I*_), *u*_*i*_(*o*′, *θ*_*i*_) > *u*_*i*_(*o*, *θ*_*i*_)→∃*j* ∈ *I*, *u*_*j*_(*o*′, *θ*_*j*_) < *u*_*j*_(*o*, *θ*_*j*_). This is a formal expression of Pareto solution. The mechanism design of EC implements an equilibrium solution (such as Nash strategy equilibrium in this paper). The desired target of mechanism design for EC is to find the optimal solution of an optimized problem. If the equilibrium solution can guarantee the Pareto optimal of an EC implementation, that is, for every individual *i*, *u*_*i*_(*o*′, *θ*_*i*_) > *u*_*i*_(*o*, *θ*_*i*_), the convergence state of an EC algorithm can be achieved in this sense. We can establish a formal framework to study on EC convergence based on the equilibrium solution implementation issue. On the other hand, If the equilibrium solution cannot guarantee a Pareto solution, we also need to implement Pareto optimal solution of EC so as to achieve the maximal optimization capability of the algorithm. In this sense, the issues of EC convergence and optimization capability can be transformed as a Pareto solution implementation issue in the algorithmic mechanism design of EC.

## 6. Conclusion and Future Work

In this paper, we proposed a formal EC framework by considering it as a game or mechanism design problem. The individuals in EC are designed as rational, self-interested agents within this framework and can play using their own search schemes with certain information defined in the game. We designed this framework by defining agent, type, strategy, payoff, and other concepts in EC algorithm. As the first step, we simplified the EC algorithm mechanism design problem to find the Nash strategy equilibrium of fitness improvement as the target of the problem under an assumption of a quasilinear preference of an agent and tried to find the appropriate strategy to achieve this objective. A Nash strategy equilibrium-based DE algorithm considering NE strategy of scale factor *F*, NE strategy of crossover rate Cr, NE strategy of mutation, and a mixture of these was initially implemented, analysed, and discussed. We also came to the interesting conclusion that Nash equilibrium solution is also a dominant solution in our designed algorithm.

The motivation and final objective of this study was to bring the fundamental aspects of game theory and algorithmic mechanism design into the EC field. The design, enhancement, and acceleration of an EC algorithm can therefore be treated as an algorithmic mechanism design problem. This aspect is an original contribution made by this paper. In addition, there are several research subjects that came to light and deserve further investigation. The first is to establish an EC algorithm-related entity mechanism design theory by considering the specific characteristics of evolutionary search, stochastic optimization, and metaheuristics. The second is to design more efficient and effective EC algorithms by introducing a variety of EC implementations. The third is to investigate basic implementation issues with regard to information, agent, type, and strategy in game theory and mechanism design for EC algorithm design. The fourth is to analyse and investigate the relationship between the solution concept implementation and EC convergence and compare the related work with proposals we introduced, such as [26, 27]. These are some of the topics and subjects we will examine in our future research.

## Figures and Tables

**Algorithm 1:**
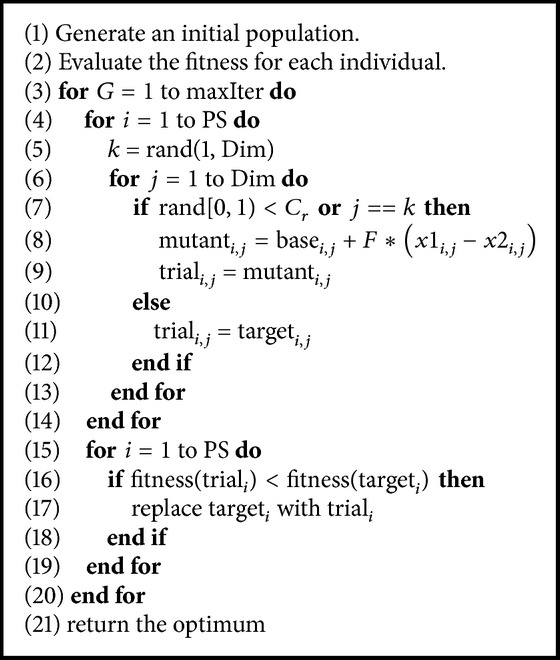
Differential evolution algorithm. PS: population size; Dim: dimension; *G*: generation; maxIter: maximum generation; *i*: index of individual; *j*: index of dimension. fitness(*∗*) is a fitness function.

**Algorithm 2:**
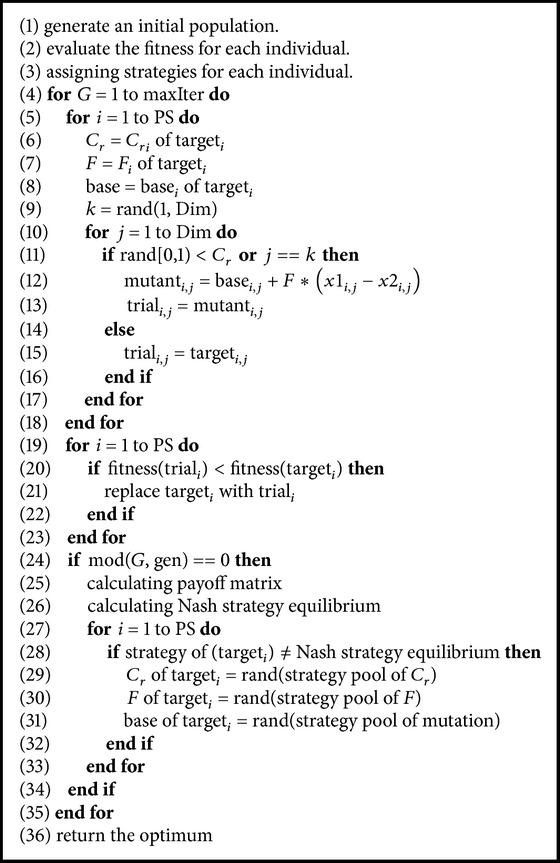
Nash strategy equilibrium-based DE algorithm. fitness(*∗*): fitness function of individual *∗*; PS: population size; Dim: dimension; *G*: generation; maxIter: maximum generation; *i*: index of individual; *j*: index of dimension; gen is jump number that controls Nash equilibrium strategy investigation by several generation; in our experimental evaluation, we set gen = 1.

**Table 1:** Payoff matrix of different strategies; Group A and Group B are composed of individuals with the same size. Strategy 1 = “DE/best/1/bin” and strategy 2 = “DE/rand/1/bin” when it is a mutation payoff matrix; strategy 1 = “1 ≥ Cr > 0.5” and strategy 2 = “0 < Cr ≤ 0.5” when it is a crossover rate payoff matrix; strategy 1 = “1 ≥ *F* > 0.5” and strategy 2 = “0 < *F* ≤ 0.5” when it is a scale factor payoff matrix. Pay_group,strategy_ presents fitness improvement (utility) of the group with the strategy.

Group B	Group A	
	Strategy 1	Strategy 2
Strategy 1	(Pay_*a*,*s*1_, Pay_*b*,*s*1_)	(Pay_*a*,*s*2_, Pay_*b*,*s*1_)
Strategy 2	(Pay_*a*,*s*1_, Pay_*b*,*s*2_)	(Pay_*a*,*s*2_, Pay_*b*,*s*2_)

**Table 2:** Benchmark functions.

Number	Type	Characteristic	Bounds	Optimum fitness
*F*1	Uni	Sh Sphere	[−100, 100]	−450
*F*2		Sh Schwefel 1.2		−450
*F*3		Sh Rt Elliptic		−450
*F*4		*F*2 with Noise		−450
*F*5		Schwefel 2.6 GB		−310

*F*6	Multi	Sh Rosenbrock	[−100, 100]	390
*F*7		Sh Rt Griewank	[0, 600]	−180
*F*8		Sh Rt Ackley GB	[−32, 32]	−140
*F*9		Sh Rastrigin	[−5, 5]	−330
*F*10		Sh Rt Rastrigin	[−5, 5]	−330
*F*11		Sh Rt Weierstrass	[−0.5, 0.5]	90
*F*12		Schwefel 2.13	[*π*, *π*]	−460
*F*13		Sh Expanded *F*8*F*2	[−3, 1]	−130
*F*14		Sh Rt Scaffer 6	[−100, 100]	−300

Uni = unimodal, Multi = multimodal, Sh = shifted, Rt = rotated, and GB = global on bounds.

**Table 3:** Algorithm parameter setting and abbreviations of the algorithms used in evaluation.

Abbreviation	Meaning
DE-best	DE with best vector as base vector.
DE-rand	DE with random vector as base vector.

jDE-best	DE-best with self-adapting control parameters method [7].
jDE-rand	DE-rand with self-adapting control parameters method [7].

Best-*F*	DE-best by selecting “1 ≥ *F* > 0.5” and “0 < *F* ≤ 0.5” as NE strategy.
Rand-*F*	DE-rand by selecting “1 ≥ *F* > 0.5” and “0 < *F* ≤ 0.5” as NE strategy.
Best-Cr	DE-best by selecting “1 ≥ Cr > 0.5” and “0 < Cr ≤ 0.5” as NE strategy.
Rand-Cr	DE-rand by selecting “1 ≥ Cr > 0.5” and “0 < Cr ≤ 0.5” as NE strategy.
Mutation	DE by selecting “best vector” and “random vector” as NE strategy.
Mixture	DE with by selecting mutation (“best vector” and “random vector”), crossover rate (“1 ≥ *F* > 0.5” and “0 < *F* ≤ 0.5”), and scale factor (“1 ≥ Cr > 0.5” and “0 < Cr ≤ 0.5”) as NE strategies.

**Table 4:** Mean and Wilcoxon signed-rank test results of 5D benchmark functions.

	DE-best	DE-rand	jDE-best	jDE-rand	Best-*F*	Rand-*F*
*F*1	9.56*E* + 03	1.17*E* + 04	3.34*E* + 03	3.34*E* + 03	1.02*E* + 04	1.15*E* + 04
*F*2	1.78*E* + 04	1.56*E* + 04	5.82*E* + 03	5.91*E* + 03	1.71*E* + 04	1.65*E* + 04
*F*3	3.46*E* + 08	6.06*E* + 08	7.43*E* + 07	7.43*E* + 07	4.08*E* + 08	3.15*E* + 08
*F*4	1.51*E* + 04	1.49*E* + 04	6.96*E* + 03	6.25*E* + 03	1.82*E* + 04	1.62*E* + 04
*F*5	8.08*E* + 03	9.33*E* + 03	5.20*E* + 03	5.20*E* + 03	1.01*E* + 04	9.73*E* + 03
*F*6	3.41*E* + 09	4.20*E* + 09	1.29*E* + 09	1.35*E* + 09	3.14*E* + 09	2.66*E* + 09‡
*F*7	8.43*E* + 01	1.95*E* + 02	−5.21*E* + 01	−5.07*E* + 01	2.44*E* + 02	2.25*E* + 02
*F*8	−1.19*E* + 02	−1.19*E* + 02	−1.19*E* + 02	−1.19*E* + 02	−1.19*E* + 02	−1.19*E* + 02†‡
*F*9	−2.63*E* + 02	−2.57*E* + 02	−2.97*E* + 02	−2.97*E* + 02	−2.63*E* + 02	−2.76*E* + 02†‡
*F*10	−2.29*E* + 02	−2.30*E* + 02	−2.84*E* + 02	−2.79*E* + 02	−2.38*E* + 02	−2.51*E* + 02†‡
*F*11	9.74*E* + 01	9.71*E* + 01	9.51*E* + 01	9.51*E* + 01	9.71*E* + 01†‡	9.61*E* + 01†‡
*F*12	4.35*E* + 04	3.30*E* + 04	1.42*E* + 04	1.41*E* + 04	3.89*E* + 04	2.60*E* + 04†
*F*13	4.34*E* + 01	4.99*E* + 01	−1.11*E* + 02	−1.11*E* + 02	−2.92*E* + 01‡§£	−6.55*E* + 01†‡§£
*F*14	−2.98*E* + 02	−2.98*E* + 02	−2.98*E* + 02	−2.98*E* + 02	−2.98*E* + 02‡	−2.98*E* + 02†‡

	Best-Cr	Rand-Cr	Mutation	Mixture	VTR	

*F*1	3.08*E* + 03†‡	3.09*E* + 03†‡	1.10*E* + 04	3.45*E* + 03†‡	7.02*E* + 03	
*F*2	6.49*E* + 03†‡	4.43*E* + 03†‡	1.53*E* + 04	6.02*E* + 03†‡	1.11*E* + 04	
*F*3	8.08*E* + 07	1.91*E* + 08†‡	3.64*E* + 08‡	1.34*E* + 08†‡	2.59*E* + 08	
*F*4	8.05*E* + 03†‡	4.70*E* + 03§	1.59*E* + 04	6.01*E* + 03†‡	1.12*E* + 04	
*F*5	4.11*E* + 03†‡	2.91*E* + 03†‡§£	8.43*E* + 03	5.44*E* + 03†‡	6.85*E* + 03	
*F*6	1.25*E* + 09†‡	7.50*E* + 08†‡	4.27*E* + 09	1.28*E* + 09†‡	2.36*E* + 09	
*F*7	−3.14*E* + 01†‡	−5.40*E* + 01†‡	9.63*E* + 01	−9.81*E* + 00†‡	6.48*E* + 01	
*F*8	−1.20*E* + 02†‡§£	−1.20*E* + 02†‡§£	−1.19*E* + 02§£	−1.20*E* + 02†‡§£	−1.19*E* + 02	
*F*9	−2.89*E* + 02†‡	−3.01*E* + 02†‡	−2.64*E* + 02‡	−3.12*E* + 02†‡§£	−2.82*E* + 02	
*F*10	−2.73*E* + 02†‡	−2.83*E* + 02†‡	−2.30*E* + 02	−2.98*E* + 02†‡§£	−2.59*E* + 02	
*F*11	9.49*E* + 01†‡	9.44*E* + 01†‡§£	9.69*E* + 01	9.32*E* + 01	9.57*E* + 01	
*F*12	1.67*E* + 04†‡	9.81*E* + 03†‡	3.57*E* + 04†	3.74*E* + 03†‡§£	2.36*E* + 04	
*F*13	−1.09*E* + 02†‡	−9.69*E* + 01†‡	1.17*E* + 01	−1.09*E* + 02†‡	−5.26*E* + 01	
*F*14	−2.98*E* + 02†‡§£	−2.98*E* + 02	−2.98*E* + 02	−2.98*E* + 02†‡§£	−2.98*E* + 02	

†, ‡, §, and £ present proposed method which is significantly better than DE-best, DE-rand, jDE-best, and jDE-rand from Wilcoxon signed-rank test (*p* < 0.05), respectively.

**Table 5:** Mean and Wilcoxon signed-rank test results of 10D benchmark functions.

	DE-best	DE-rand	jDE-best	jDE-rand	Best-*F*	Rand-*F*
*F*1	3.53*E* + 04	3.97*E* + 04	1.32*E* + 04	1.29*E* + 04	3.39*E* + 04‡	3.26*E* + 04‡
*F*2	4.86*E* + 04	4.70*E* + 04	1.65*E* + 04	1.66*E* + 04	4.89*E* + 04	4.30*E* + 04†
*F*3	9.82*E* + 08	1.04*E* + 09	2.54*E* + 08	2.42*E* + 08	8.45*E* + 08	7.81*E* + 08
*F*4	5.13*E* + 04	4.94*E* + 04	1.71*E* + 04	1.94*E* + 04	5.05*E* + 04	3.92*E* + 04†‡
*F*5	2.36*E* + 04	2.43*E* + 04	1.53*E* + 04	1.52*E* + 04	2.37*E* + 04	2.36*E* + 04
*F*6	3.26*E* + 10	3.20*E* + 10	9.99*E* + 09	9.76*E* + 09	2.55*E* + 10†‡	2.26*E* + 10†‡
*F*7	1.50*E* + 03	1.60*E* + 03	3.11*E* + 02	3.11*E* + 02	1.45*E* + 03‡	1.37*E* + 03‡
*F*8	−1.19*E* + 02	−1.19*E* + 02	−1.19*E* + 02	−1.19*E* + 02	−1.19*E* + 02	−1.19*E* + 02‡
*F*9	−1.58*E* + 02	−1.58*E* + 02	−2.39*E* + 02	−2.37*E* + 02	−1.74*E* + 02†‡	−1.91*E* + 02†‡
*F*10	−6.99*E* + 01	−6.92*E* + 01	−1.95*E* + 02	−2.00*E* + 02	−8.97*E* + 01†‡	−1.11*E* + 02†‡
*F*11	1.06*E* + 02	1.06*E* + 02	1.02*E* + 02	1.02*E* + 02	1.05*E* + 02	1.04*E* + 02†‡
*F*12	2.95*E* + 05	3.39*E* + 05	9.34*E* + 04	9.54*E* + 04	3.17*E* + 05	2.55*E* + 05†‡
*F*13	7.40*E* + 02	7.55*E* + 02	−1.22*E* + 01	−1.21*E* + 01	4.88*E* + 02†‡	4.14*E* + 02†‡
*F*14	−2.95*E* + 02	−2.95*E* + 02	−2.96*E* + 02	−2.96*E* + 02	−2.95*E* + 02	−2.95*E* + 02

	Best-Cr	Rand-Cr	Mutation	Mixture	VTR	

*F*1	1.17*E* + 04†‡	8.30*E* + 03†‡§£	3.79*E* + 04	1.68*E* + 04†‡	2.42*E* + 04	
*F*2	1.46*E* + 04†‡	1.21*E* + 04†‡§£	4.49*E* + 04	1.84*E* + 04†‡	3.11*E* + 04	
*F*3	2.70*E* + 08†‡	8.90*E* + 07†‡§£	7.62*E* + 08‡	2.54*E* + 08†‡	5.52*E* + 08	
*F*4	1.47*E* + 04†‡	1.61*E* + 04	4.52*E* + 04	2.07*E* + 04†‡	3.24*E* + 04	
*F*5	1.03*E* + 04†‡§£	1.06*E* + 04†‡§£	2.30*E* + 04§£	1.49*E* + 04†‡	1.85*E* + 04	
*F*6	6.80*E* + 09†‡§£	3.97*E* + 09†‡§£	3.15*E* + 10	1.21*E* + 10†‡	1.87*E* + 10	
*F*7	3.63*E* + 02†‡	1.51*E* + 02†‡§£	1.52*E* + 03	6.08*E* + 02†‡	9.19*E* + 02	
*F*8	−1.19*E* + 02†‡§£	−1.19*E* + 02†‡§£	−1.19*E* + 02	−1.19*E* + 02†‡§£	−1.19*E* + 02	
*F*9	−2.50*E* + 02†‡	−2.48*E* + 02	−1.63*E* + 02	−2.56*E* + 02†‡£	−2.07*E* + 02	
*F*10	−1.96*E* + 02†‡	−2.33*E* + 02†‡§£	−7.75*E* + 01	−2.16*E* + 02†‡	−1.46*E* + 02	
*F*11	1.01*E* + 02†‡§£	9.98*E* + 01	1.06*E* + 02	9.93*E* + 01†‡§£	1.03*E* + 02	
*F*12	6.99*E* + 04†‡£	6.44*E* + 04†‡§£	2.99*E* + 05	6.83*E* + 04†‡§£	1.90*E* + 05	
*F*13	1.65*E* + 01†‡	−8.43*E* + 01†‡	5.29*E* + 02	4.83*E* + 01†‡	2.88*E* + 02	
*F*14	−2.96*E* + 02†‡§£	−2.96*E* + 02†‡§£	−2.95*E* + 02	−2.96*E* + 02†‡§£	−2.95*E* + 02	

†, ‡, §, and £ are with the same meaning as in Table 4.

**Table 6:** Mean and Wilcoxon signed-rank test results of 30D benchmark functions.

	DE-best	DE-rand	jDE-best	jDE-rand	Best-*F*	Rand-*F*
*F*1	1.45*E* + 05	1.44*E* + 05	7.99*E* + 04	8.07*E* + 04	1.23*E* + 05†‡	1.17*E* + 05†‡
*F*2	4.71*E* + 05	4.41*E* + 05	1.04*E* + 05	1.06*E* + 05	4.86*E* + 05	4.26*E* + 05
*F*3	5.59*E* + 09	5.92*E* + 09	1.39*E* + 09	1.41*E* + 09	4.64*E* + 09†‡	4.01*E* + 09†‡
*F*4	4.28*E* + 05	4.42*E* + 05	1.22*E* + 05	1.54*E* + 05	4.92*E* + 05	4.39*E* + 05
*F*5	5.62*E* + 04	5.50*E* + 04	3.81*E* + 04	3.80*E* + 04	5.66*E* + 04	5.56*E* + 04
*F*6	1.89*E* + 11	1.87*E* + 11	6.99*E* + 10	7.01*E* + 10	1.33*E* + 11†‡	1.02*E* + 11†‡
*F*7	5.91*E* + 03	6.04*E* + 03	2.93*E* + 03	2.92*E* + 03	5.52*E* + 03†‡	5.30*E* + 03†‡
*F*8	−1.18*E* + 02	−1.19*E* + 02	−1.19*E* + 02	−1.19*E* + 02	−1.19*E* + 02	−1.19*E* + 02†
*F*9	3.38*E* + 02	3.30*E* + 02	1.09*E* + 02	1.06*E* + 02	2.69*E* + 02†‡	2.46*E* + 02†‡
*F*10	8.48*E* + 02	8.80*E* + 02	4.33*E* + 02	4.38*E* + 02	7.61*E* + 02†‡	6.66*E* + 02†‡
*F*11	1.45*E* + 02	1.43*E* + 02	1.33*E* + 02	1.33*E* + 02	1.43*E* + 02†	1.42*E* + 02†
*F*12	3.12*E* + 06	3.06*E* + 06	1.25*E* + 06	1.28*E* + 06	2.92*E* + 06	2.72*E* + 06†‡
*F*13	6.27*E* + 03	6.05*E* + 03	8.76*E* + 02	8.75*E* + 02	3.75*E* + 03†‡	1.85*E* + 03†‡
*F*14	−2.85*E* + 02	−2.85*E* + 02	−2.86*E* + 02	−2.86*E* + 02	−2.85*E* + 02†	−2.85*E* + 02†‡

	Best-Cr	Rand-Cr	Mutation	Mixture	VTR	

*F*1	6.40*E* + 04†‡§£	4.63*E* + 04†‡§£	1.41*E* + 05	7.40*E* + 04†‡	1.02*E* + 05	
*F*2	1.39*E* + 05†‡	8.57*E* + 04†‡§£	3.86*E* + 05	1.25*E* + 05†‡	2.77*E* + 05	
*F*3	1.03*E* + 09†‡	5.60*E* + 08†‡§£	5.34*E* + 09	1.50*E* + 09	3.14*E* + 09	
*F*4	1.46*E* + 05†‡	8.47*E* + 04†‡§£	3.59*E* + 05	1.54*E* + 05†‡	2.82*E* + 05	
*F*5	3.09*E* + 04†‡§£	2.42*E* + 04†‡§£	5.35*E* + 04†	3.61*E* + 04†‡	4.44*E* + 04	
*F*6	7.25*E* + 10†‡	3.49*E* + 10†‡§£	1.83*E* + 11	6.44*E* + 10†‡	1.11*E* + 11	
*F*7	2.50*E* + 03†‡	1.79*E* + 03†‡§£	5.85*E* + 03	2.85*E* + 03†‡	4.16*E* + 03	
*F*8	−1.19*E* + 02†‡§£	−1.19*E* + 02†‡§£	−1.19*E* + 02	−1.19*E* + 02†‡§£	−1.19*E* + 02	
*F*9	7.53*E* + 01†‡	−1.73*E* + 01†‡§£	3.51*E* + 02	1.09*E* + 01†‡§£	1.82*E* + 02	
*F*10	2.53*E* + 02†‡§£	1.62*E* + 02†‡§£	8.39*E* + 02	3.15*E* + 02†‡§£	5.59*E* + 02	
*F*11	1.30*E* + 02†‡§£	1.29*E* + 02†‡§£	1.43*E* + 02	1.26*E* + 02†‡§£	1.37*E* + 02	
*F*12	1.27*E* + 06†‡	6.94*E* + 05†‡§£	3.07*E* + 06	7.18*E* + 05†‡§£	2.01*E* + 06	
*F*13	1.36*E* + 03†‡	8.21*E* + 02†‡§£	5.97*E* + 03	9.18*E* + 02†‡	2.87*E* + 03	
*F*14	−2.86*E* + 02	−2.86*E* + 02	−2.85*E* + 02	−2.86*E* + 02†‡	−2.86*E* + 02	

†, ‡, §, and £ are with the same meaning as in Table 4.

**(a):** 

Function	DE-best		DE-rand			jDE-best			jDE-rand			Best-*F*		
	NFC	SR	NFC	SR	AR	NFC	SR	AR	NFC	SR	AR	NFC	SR	AR
*F*1	19022	0.37	19024	0.37	97.04	3132	0.90	219.20	3132	0.90	219.20	19018	0.37	113.83
*F*2	18021	0.40	17032	0.43	38.28	4160	0.86	157.48	4160	0.86	157.48	19012	0.37	148.01
*F*3	14025	0.53	17041	0.43	40.80	4077	0.86	238.19	4079	0.86	238.17	15022	0.50	153.89
*F*4	17037	0.43	15058	0.50	39.49	6195	0.79	127.66	5297	0.82	54.31	20021	0.33	46.58
*F*5	16019	0.47	22021	0.27	0.71	8115	0.73	88.14	8126	0.73	86.84	22009	0.27	74.67
*F*6	13014	0.57	15016	0.50	20.39	5058	0.83	218.61	5050	0.83	218.70	10015	0.67	171.82
*F*7	14022	0.53	17023	0.43	70.01	4094	0.86	205.57	4094	0.86	205.57	20004	0.33	83.92
*F*8	26007	0.13	26020	0.13	22.06	13248	0.56	63.47	12219	0.59	71.18	27016	0.10	10.02
*F*9	24009	0.20	27000	0.10	0.89	5158	0.83	161.35	5158	0.83	161.35	21010	0.30	130.37
*F*10	22005	0.27	22006	0.27	40.82	6112	0.80	171.47	7109	0.76	179.88	21006	0.30	100.82
*F*11	26003	0.13	25005	0.17	31.75	8302	0.72	137.37	7284	0.76	182.02	23010	0.23	77.98
*F*12	22001	0.27	17019	0.43	135.48	7138	0.76	140.67	7138	0.76	140.67	19007	0.37	142.09
*F*13	14008	0.53	16004	0.47	27.16	2072	0.93	193.09	2072	0.93	193.09	9016	0.70	237.42
*F*14	28006	0.07	29001	0.03	34.23	14184	0.53	182.18	14191	0.53	181.66	25013	0.17	105.53

Average	19514.21	0.35	20305.00	0.32	42.79	6503.21	0.78	164.60	6364.93	0.79	163.58	19298.50	0.36	114.07

**(b):** 

Function	Rand-*F*			Best-Cr			Rand-Cr			Mutation			Mixture		
	NFC	SR	AR	NFC	SR	AR	NFC	SR	AR	NFC	SR	AR	NFC	SR	AR
*F*1	25015	0.17	0.72	3152	0.89	164.36	4177	0.86	107.04	19016	0.37	90.20	3367	0.89	208.62
*F*2	20016	0.33	71.13	6215	0.79	82.35	4539	0.85	76.96	19023	0.37	51.93	7137	0.76	170.13
*F*3	14030	0.53	112.95	1156	0.96	153.02	2783	0.91	54.13	15018	0.50	74.25	4159	0.86	149.96
*F*4	19046	0.37	26.03	10177	0.66	48.29	3978	0.87	45.09	22050	0.27	0.72	5513	0.82	97.85
*F*5	22021	0.27	45.04	4235	0.86	103.76	3255	0.89	98.19	17034	0.43	103.63	10295	0.66	73.34
*F*6	10032	0.67	116.85	5054	0.83	161.90	3103	0.90	148.66	13014	0.57	70.79	3143	0.90	158.03
*F*7	17020	0.43	59.56	2114	0.93	165.37	4128	0.86	160.04	13028	0.57	215.68	7142	0.76	151.00
*F*8	17075	0.43	109.19	4795	0.84	65.60	2236	0.93	98.39	26010	0.13	50.79	715	0.98	128.44
*F*9	15058	0.50	118.20	10177	0.66	100.23	4248	0.86	99.42	22010	0.27	96.46	1496	0.95	135.55
*F*10	15031	0.50	166.54	7174	0.76	102.39	3632	0.88	95.18	22014	0.27	34.09	5221	0.83	219.77
*F*11	16083	0.46	80.30	7441	0.75	132.90	4907	0.84	161.32	26001	0.13	37.96	2121	0.93	95.21
*F*12	17023	0.43	84.50	6358	0.79	110.48	3371	0.89	122.79	21009	0.30	20.43	1243	0.96	179.52
*F*13	8038	0.73	206.99	3072	0.90	217.49	1159	0.96	161.38	10040	0.67	152.20	2106	0.93	275.64
*F*14	22046	0.27	94.53	7069	0.76	72.58	4927	0.84	82.05	28009	0.07	5.75	3285	0.89	137.22

Average	16966.71	0.43	92.32	5584.93	0.81	120.05	3603.07	0.88	107.90	19519.71	0.35	71.78	4067.36	0.86	155.73

**(a):** 

Function	DE-best		DE-rand			jDE-best			jDE-rand			Best-*F*		
	NFC	SR	NFC	SR	AR	NFC	SR	AR	NFC	SR	AR	NFC	SR	AR
*F*1	27005	0.10	28001	0.07	0.97	2230	0.93	207.14	2244	0.93	203.11	23010	0.23	104.34
*F*2	25008	0.17	24017	0.20	24.01	2246	0.93	217.86	2252	0.92	187.24	22009	0.27	152.54
*F*3	13032	0.57	20019	0.33	33.80	3135	0.90	103.28	3135	0.90	103.26	15020	0.50	71.44
*F*4	24029	0.20	23064	0.23	12.14	3237	0.89	162.18	6170	0.79	136.74	23010	0.23	84.30
*F*5	25004	0.17	25002	0.17	20.15	6211	0.79	113.41	5252	0.82	114.31	24006	0.20	13.79
*F*6	17010	0.43	19006	0.37	23.56	5143	0.83	115.89	5148	0.83	99.66	15016	0.50	85.78
*F*7	23013	0.23	28000	0.07	0.82	2156	0.93	235.93	2158	0.93	235.89	23007	0.23	107.96
*F*8	29002	0.03	27028	0.10	10.63	3725	0.88	100.05	2760	0.91	83.58	29004	0.03	9.27
*F*9	28002	0.07	28010	0.07	4.38	7161	0.76	160.58	7163	0.76	160.22	28006	0.07	6.68
*F*10	26005	0.13	24020	0.20	18.55	5156	0.83	183.77	4161	0.86	191.14	23014	0.23	53.28
*F*11	30000	0.00	29004	0.03	9.30	6548	0.78	57.28	7522	0.75	56.03	30000	0.00	1.00
*F*12	24005	0.20	25005	0.17	47.11	2217	0.93	204.25	2217	0.93	177.32	26001	0.13	37.89
*F*13	19003	0.37	17008	0.43	59.13	3089	0.90	287.70	3089	0.90	287.70	9017	0.70	383.93
*F*14	29000	0.03	28005	0.07	7.86	12446	0.59	60.56	12485	0.58	56.88	27003	0.10	52.66

Average	24222.71	0.19	24656.36	0.18	19.46	4621.43	0.85	157.85	4696.86	0.84	149.50	22651.64	0.24	83.20

**(b):** 

Function	Rand-*F*			Best-Cr			Rand-Cr			Mutation			Mixture		
	NFC	SR	AR	NFC	SR	AR	NFC	SR	AR	NFC	SR	AR	NFC	SR	AR
*F*1	22015	0.27	138.67	5348	0.82	199.52	3524	0.88	178.57	27012	0.10	12.68	8176	0.73	185.79
*F*2	17038	0.43	126.74	1472	0.95	254.04	2215	0.93	220.01	20020	0.33	90.14	6249	0.79	198.93
*F*3	14036	0.53	69.17	2202	0.93	153.31	1341	0.96	170.20	15025	0.50	122.34	4389	0.85	91.31
*F*4	15110	0.50	97.05	850	0.97	139.85	3131	0.90	131.02	21073	0.30	73.46	6235	0.79	158.21
*F*5	25010	0.17	4.77	4521	0.85	109.69	3436	0.89	87.99	22029	0.27	34.72	10193	0.66	102.39
*F*6	13030	0.57	102.26	3259	0.89	170.81	2330	0.92	164.54	18011	0.40	8.58	7065	0.76	187.85
*F*7	23024	0.23	19.74	4283	0.86	190.52	388	0.99	209.71	26003	0.13	54.32	8208	0.73	96.44
*F*8	27026	0.10	13.16	6050	0.80	118.38	3335	0.89	89.72	28006	0.07	40.90	1855	0.94	110.37
*F*9	21054	0.30	53.37	5426	0.82	146.13	4463	0.85	151.74	28001	0.07	35.41	5289	0.82	129.25
*F*10	20040	0.33	50.98	5475	0.82	196.59	2560	0.91	186.79	25009	0.17	19.41	5329	0.82	158.76
*F*11	25034	0.17	35.66	5344	0.82	68.75	1825	0.94	74.58	29005	0.03	7.63	3585	0.88	112.46
*F*12	20037	0.33	70.29	3325	0.89	124.09	2442	0.92	109.70	24016	0.20	35.01	3395	0.89	151.15
*F*13	10027	0.67	274.26	2287	0.92	138.07	233	0.99	187.59	17007	0.43	67.47	3074	0.90	405.78
*F*14	26039	0.13	8.94	4640	0.85	43.96	4574	0.85	39.66	29000	0.03	1.00	3355	0.89	53.80

Average	19894.29	0.34	76.08	3891.57	0.87	146.69	2556.93	0.91	142.99	23515.5	0.22	43.08	5456.93	0.82	153.03

**(a):** 

Function	DE-best		DE-rand			jDE-best			jDE-rand			Best-*F*		
	NFC	SR	NFC	SR	AR	NFC	SR	AR	NFC	SR	AR	NFC	SR	AR
*F*1	29003	0.03	30000	0.00	0.97	4288	0.86	115.72	4297	0.86	109.95	24017	0.20	75.77
*F*2	22010	0.27	21020	0.30	43.66	154	0.99	281.16	156	0.99	271.54	19022	0.37	154.49
*F*3	25003	0.17	26000	0.13	0.96	2209	0.93	181.63	2210	0.93	178.42	25003	0.17	13.74
*F*4	24007	0.20	21028	0.30	44.54	210	0.99	207.12	3403	0.89	236.47	19144	0.36	101.97
*F*5	27002	0.10	28011	0.07	4.03	6253	0.79	113.75	6253	0.79	113.75	25007	0.17	78.45
*F*6	29000	0.03	28003	0.07	12.46	4198	0.86	232.62	4201	0.86	222.27	16022	0.47	342.51
*F*7	27001	0.10	28000	0.07	0.96	6143	0.80	193.45	6145	0.80	192.85	22015	0.27	148.07
*F*8	27002	0.10	27007	0.10	25.00	5574	0.81	51.12	6092	0.80	51.65	26008	0.13	59.45
*F*9	30000	0.00	29005	0.03	7.63	5290	0.82	98.63	5289	0.82	99.09	24018	0.20	110.52
*F*10	28000	0.07	28000	0.07	1.00	8306	0.72	98.62	8313	0.72	84.73	25008	0.17	42.56
*F*11	30000	0.00	29007	0.03	5.73	3945	0.87	49.77	3967	0.87	47.05	30000	0.00	1.00
*F*12	29003	0.03	29003	0.03	12.04	1428	0.95	83.80	1433	0.95	81.82	27008	0.10	57.53
*F*13	25003	0.17	27000	0.10	0.93	1136	0.96	343.75	1136	0.96	343.75	13020	0.57	432.51
*F*14	30000	0.00	30000	0.00	1.00	5502	0.82	38.63	6391	0.79	38.28	27010	0.10	49.23

Average	27288.14	0.09	27220.29	0.09	11.49	3902.57	0.87	149.27	4234.71	0.86	147.97	23021.57	0.23	119.13

**(b):** 

Function	Rand-*F*			Best-Cr			Rand-Cr			Mutation			Mixture		
	NFC	SR	AR	NFC	SR	AR	NFC	SR	AR	NFC	SR	AR	NFC	SR	AR
*F*1	22043	0.27	68.08	8270	0.72	77.93	3482	0.88	67.51	29003	0.03	12.04	5316	0.82	99.79
*F*2	16042	0.47	147.32	4166	0.86	208.80	1239	0.96	163.57	18034	0.40	92.14	3157	0.89	264.24
*F*3	18037	0.40	85.96	2282	0.92	164.60	2291	0.92	119.83	24003	0.20	58.58	5269	0.82	148.67
*F*4	15097	0.50	103.86	4301	0.86	229.62	440	0.99	139.78	18046	0.40	83.75	4174	0.86	244.87
*F*5	27007	0.10	7.81	5393	0.82	110.24	1471	0.95	69.04	28005	0.07	0.94	6945	0.77	83.91
*F*6	8074	0.73	298.02	9213	0.69	98.24	2314	0.92	125.94	29000	0.03	1.00	6199	0.79	207.02
*F*7	24012	0.20	67.49	5352	0.82	117.66	1335	0.96	124.71	27002	0.10	18.79	5241	0.83	141.25
*F*8	22068	0.26	31.45	3956	0.87	44.36	2043	0.93	49.22	28014	0.07	3.36	2442	0.92	55.25
*F*9	27015	0.10	21.46	10326	0.66	53.76	2652	0.91	63.02	30000	0.00	1.00	2477	0.92	81.18
*F*10	20045	0.33	55.26	5381	0.82	85.14	590	0.98	87.82	27004	0.10	9.89	6346	0.79	128.95
*F*11	29001	0.03	34.30	4624	0.85	26.51	1855	0.94	29.82	30000	0.00	1.00	1255	0.96	71.54
*F*12	26034	0.13	28.18	7394	0.75	70.51	2470	0.92	67.68	29001	0.03	34.27	2471	0.92	69.65
*F*13	7059	0.76	330.94	6209	0.79	240.19	1383	0.95	266.84	26002	0.13	19.41	1200	0.96	286.63
*F*14	27033	0.10	10.85	4665	0.84	36.55	4050	0.87	22.36	30000	0.00	1.00	3510	0.88	30.67

Average	20611.93	0.31	92.21	5823.71	0.80	111.72	1972.50	0.93	99.79	26651	0.11	24.08	4000.14	0.86	136.68
